# Effect of yeast fermentation on nutrient utilization and growth performance of broilers fed commercial and hand-mixed diets

**DOI:** 10.5455/javar.2026.m1016

**Published:** 2026-03-10

**Authors:** Syful Islam, Saima Siddique, Sharmila Islam, Jesmin Aktar, Momota Rani Debi, Khan Md. Shaiful Islam

**Affiliations:** 1 Department of Animal Nutrition, Bangladesh Agricultural University, Mymensingh-2202, Bangladesh

**Keywords:** Fermentation, feeding, nutrition, additive, synthetic amino acid

## Abstract

**Objectives:**

This study aimed to evaluate the effects of fermentation efficiency, nutritional components, digestibility, and growth performance between commercial and hand-mixed diets.

**Materials and Methods:**

In the first trial, 144 Cobb 308-day-old chicks were distributed into three groups, with six replications and eight birds per replicate cage. They were offered a commercial dry diet (CD), a moist commercial diet (CM), and a fermented commercial moist diet (CFM). In the second trial, another 144 Cobb 308-day-old chicks were grouped in threes (similarly distributed) and offered a hand-mixed moist diet (HM), a fermented hand-mixed moist diet (HFM), and a fermented hand mixed moist diet without synthetic amino acids (HFM-SAA). Feed composition, apparent nutrient digestibility, growth indices, and economic returns were analyzed for 35 days feeding trial.

**Results:**

The fermentation effect was non-significant on nutrient composition and energy content in the first trial, but reduced feed intake, final body weight (*p* < 0.001), and profit (*p* = 0.001). In the second trial, fermentation significantly increased crude protein (*p* = 0.002), dry matter and protein digestibility (*p* < 0.01), and body weight gain (*p* < 0.001), resulting in higher profit in the fermented feeding group. Removing synthetic amino acids neglects these benefits. In comparison, trial one achieved better outcomes in the non-fermented state, while trial two achieved better outcomes in the fermented group after adding synthetic amino acids.

**Conclusions:**

Fermentation benefits are feed-type dependent, favoring a simple hand-mixed diet. Absence of synthetic amino acids also demonstrates a negative impact.

## 1. Introduction

The global demand for animal protein continues to rise, with poultry meat signifying a major share of total consumption due to its affordability, versatility, and broad cultural acceptance. Global poultry consumption is projected to reach 173 MT ready-to-cook (RTC) by 2034, accounting for 62% of the additional meat consumed globally [1], replicating its role as one of the most rapidly expanding animal protein sources. Broiler production has become one of the fastest-growing sectors in animal agriculture, largely because chicken meat is free from cultural or religious restrictions and is widely consumed across regions [2]. This growth has been complemented by challenges, as the industry must maintain efficiency while addressing consumer concerns about food safety, animal welfare, and environmental sustainability [3, 4]. Feed remains the largest contributor to production costs, accounting for up to 70% of total expenses [5, 6]. Feed prices are rising daily amid restrictions on the use of antibiotic growth promoters in animal feed worldwide. There is a strong interest in feed innovations that can improve efficiency while reducing reliance on synthetic additives [7, 8, 9].

Though dry feed in the form of mash or pellet is widely accepted in commercial poultry production, sometimes broilers show their preferences for wet feed over dry [10], where the effect of commercial moist feed is still mysterious. Besides, feed fermentation with *Saccharomyces cerevisiae* is another focus for researchers. Fermentation can improve the chemical composition of feed by increasing crude protein (CP), reducing crude fiber (CF) [11, 12], lowering anti-nutritional factors (ANFs), and increasing available nutrients [13, 14, 15]. Findings from some researchers suggest that fermented feed can be used as an alternative to conventional feed additives, with potential benefits for growth performance, digestibility, and economic benefits in broilers [16, 17, 18]. At high additive concentrations, fermentation can be inhibited by reduced microbial growth [19], indicating lower fermentation capacity in commercial feeds formulated with many additives [20]. Another study noted limited benefits when commercial diets already fortified with additives were fermented [21].

The response of broilers to fermented feed, however, is not uniform. Previous studies have demonstrated that moist feeding can enhance feed intake, weight gain, and feed conversion efficiency, particularly in hot climates or when using wheat-based diets [22]. Fermentation builds on this by introducing microbial activity, but outcomes differ depending on diet type. Several reports found improved growth and nutrient utilization with fermented feeds [18, 21]. Additionally, fermented feeds are gaining attention in poultry farming as a potential means to improve both growth performance and gut health in broilers [16, 23]. Fermented feed has considerable potential as a high-quality protein source in animal production [24], thereby reducing the need for synthetic amino acids during fermentation.

Besides biological performance, fermentation has economic potential by reducing the feed conversion ratio (FCR) and increasing profit per kg of live weight production [25]. Al-Khalaifah et al. [22] reported that fermented by-products, such as brewer’s dried grains and rice bran, can minimize feed costs without compromising performance and support more sustainable poultry production. However, the comparison between commercial and hand-mixed diets under fermentation is unknown. Considering this knowledge gap, this study tested whether yeast fermentation improves nutrient digestibility, growth, and profitability differently in complex commercial versus simple hand-mixed diets.

## 2. Materials and Methods

### 2.1. Ethical approval

The Animal Welfare and Experimental Ethics Committee of Bangladesh Agricultural University reviewed and approved the experimental protocols, animal care, and sample collection procedures. [AWEEC/BAU/2025(2)/66(a)].

### 2.2. Trial-01

#### 2.2.1. Experimental setup

A total of 144 day-old Cobb 308 broiler chicks were used in a 35-day feeding trial. During the first week, all chicks received a commercial pre-starter crumble diet to allow adaptation. At eight days of age, the birds were randomly assigned to groups, with each group consisting of 6 replicates of 8 birds (6 replicates × 8 birds per cage).

#### 2.2.2. Treatment group, fermentation procedure, and vaccination

This trial tested commercial diets under three groups: a standard commercial diet (CD), the same diet with 40% added water (CM), and a fermented commercial moist diet (CFM) where fermentation was performed using *S. cerevisiae* at 2.0% (w/w) with 40% water addition under anaerobic conditions in airtight plastic bags at 29–31°C for 48 h. Fresh feed was prepared daily and offered immediately to ensure moisture consistency. Fermentation quality was verified by pH measurement before and after fermentation, and only fresh feed with a normal fermented odor was used. Vaccination was done on the 4^th^ and 18^th^ days against Newcastle disease, and the infectious bursal disease vaccine was administered on the 11^th^ and 22^nd^ days.

### 2.3. Trial-02

#### 2.3.1. Experimental setup

The second trial was also conducted for 35 days by using 144 day-old Cobb 308 broiler chicks and followed the same adaptation period and grouping method as in Trial 01.

### 2.3.2. Feed formulation, treatment group, and fermentation procedure

This trial tested hand-mixed diets formulated with maize, soybean meal, rice bran, dicalcium phosphate (DCP), oil, limestone, salt, lysine, and methionine, each at their respective nutritional levels (Table 1). The groups were a hand-mixed moist diet (HM), a fermented moist diet prepared under the same fermentation conditions used in Trial 01 (HFM), and a fermented hand-mixed moist diet without synthetic amino acids (HFM-SAA). The yeast used for fermentation (*S. cerevisiae*) was obtained from Angel Yeast Co., Ltd., China. Feed and water were supplied *ad libitum* throughout the trial. The vaccination program was the same as that of Trial-01.

**Table 1.** Composition of the hand-mixed diet and the hand-mixed diet without synthetic amino acids.

Ingredients	HM	HM-SAA	Composition	HM	HM-SAA
Maize	48.4	48.4	ME (kcal/kg)	3426	3411
Soybean meal	39	39	CP	22.8	22.6
Rice Bran	6.0	6.0	CF	7.0	6.4
DCP	0.5	0.7	Ca	0.80	0.85
Oil	4.0	4.0	Avl. P	0.52	0.56
Limestone	1.8	1.8			
Salt	0.1	0.1			
Met	0.1	–			
Lysine	0.1	–			
Total	100	100			

ME = metabolic energy; CP = Crude Protein; CF = Crude Fiber; Ca = Calcium; Avl.P = available Phosphorus.

### 2.4. Data collection

Feed samples were analyzed for proximate composition, such as Crude Protein (CP), Crude Fiber (CF), Ether Extract (EE), Ash, and Nitrogen Free Extract (NFE), following [26] methods, and Metabolizable Energy (ME) was calculated using the formula by Adeyina et al. [27] for both trials. For the fermented diet, pH was measured before and after fermentation to confirm microbial activity. Growth performance was evaluated using initial body weight (IBW), final body weight (FBW), body weight gain (BWG), average daily gain (ADG), feed intake (FI), and feed conversion ratio (FCR). Apparent nutrient digestibility was measured on days 31–33 using acid-insoluble ash (AIA) as an internal marker, as described by Vogtmann et al. [28]. Excreta samples were collected and analyzed to determine the digestibility of dry matter (DM), crude protein (CP), crude fiber (CF), and ether extract (EE). The economic evaluation was based on prevailing market prices for feed ingredients, yeast, and chicks and included feed and production costs, as well as profit per kilogram of live weight gain.

### 2.5. Statistical analysis

Data were analyzed in a completely randomized design (CRD) using one-way ANOVA by IBM SPSS Statistics v20.0 (IBM Corp.), with significance determined at the 5% level (*p* < 0.05), and post hoc comparisons were evaluated by Tukey’s HSD test.

## 3. Results

### 3.1. Trial-01 (Commercial diet)

The nutritional composition of CD (Table 2) showed no significant differences among treatments, with minimal numerical variation. CP content was not significantly different among groups at 24.7%, 24.5%, and 24.1% for CD, CM, and CFM, respectively (*p* = 0.181). CF showed minimal variation in the statistical value, with non-significant differences (3.6%, 3.6%, and 3.7%; *p* = 0.506), and EE remained nearly constant (7.3%, 7.2%, and 7.1%; *p* = 0.811). Ash content did not differ significantly between CD (8.3%) and CFM (7.98%; *p* = 0.326), while nitrogen-free extract (NFE) marginally increased from 56.2% to 57.1% (*p* = 0.269), but the difference remained non-significant. Metabolizable energy (ME) remained statistically non-significant at 3504, 3502, and 3499 kcal/kg (*p* = 0.963).

**Table 2.** Proximate Composition of Commercial Diet (gm/100 gm DM).

Parameters (%)	CD	CM	CFM	*p*-value
Crude protein	24.7 ± 0.3	24.5 ± 0.6	24.1 ± 0.2	0.181
Crude fiber	3.6 ± 0.2	3.6 ± 0.1	3.7 ± 0.2	0.506
Ether extract	7.3 ± 0.4	7.2 ± 0.2	7.1 ± 0.3	0.811
Ash	8.3 ± 0.2	8.2 ± 0.3	7.98 ± 0.1	0.326
NFE	56.2 ± 0.7	56.6 ± 0.8	57.1 ± 0.4	0.269
ME (kcal/kg)	3504 ± 19	3502 ± 14	3499 ± 21	0.963

CD = commercial diet, CM = commercial moist diet, CFM = commercial fermented moist diet, NFE = nitrogen-free extract, and ME = metabolic energy; (mean ± SD); SD = standard deviation.

Growth performance exhibits highly significant differences (*p* < 0.001) among treatments (Table 3). In the CD, the highest FBW of 2120 gm was achieved, followed by 1925 gm in CM and 1821 gm in CFM, showing highly significant variation (*p* < 0.001). ADWG was also highly significant, with the highest in CD (71.4 gm/day), intermediate in CM (64.5 gm/day), and lowest in CFM (60.7 gm/day). FI declined significantly (*p* < 0.001) from 2988 gm in CD to 2738 gm in CFM, while FCR increased from 1.50 to 1.60 (*p* = 0.006).

**Table 3.** Effect of feeding a yeast-fermented commercial diet on growth performance and feed efficiency (*n* = 48).

Parameters (%)	CD	CM	CFM	*p*-value
IBW	120 ± 1.0	120 ± 1	119 ± 1	0.402
FBW	2120^a^ ± 18.2	1925^b^ ± 20.4	1821^c^ ± 16.5	< 0.001
BWG	2000^a^ ± 17.8	1805^b^ ± 20.4	1702^c^ ± 16	< 0.001
ADWG	71.4^a^ ± 0.6	64.5^b^ ± 0.7	60.7^c^ ± 0.6	< 0.001
FI	2988^a^ ± 24	2762^b^ ± 50.5	2738^b^ ± 8	< 0.001
FCR	1.50^b^ ± 0.0	1.53^b^ ± 0.1	1.6^a^ ± 0.0	0.006

^a, b, c^ The differences between the averages shown with different superscripts on the same line are statistically significant. IBW = initial body weight; FBW = final body weight; BWG = body weight gain; ADWG = average daily weight gain; FI = feed intake (kg); FCR = feed conversion ratio (mean ± SD).

Digestibility coefficients (Table 4) exhibited that DM digestibility was significantly higher in CD (85%) than in CFM (81%; *p* = 0.022), while EE digestibility decreased significantly from 78% (CD) and 77% (CM) to 70% in CFM (*p* = 0.019). Crude protein (CP) and crude fiber (CF) digestibility were not statistically different among treatments (*p* > 0.05).

**Table 4.** DM, CP, CF, and EE digestibility of commercial diet.

Parameters (%)	CD	CM	CFM	*p*-value
Digestibility DM	85^a^ ± 1	83^ab^ ± 1	81^b^ ± 1	0.022
Digestibility CP	82 ± 1	82 ± 1	81 ± 0	0.240
Digestibility CF	14 ± 4	13 ± 2	10 ± 2	0.330
Digestibility EE	78^a^ ± 4	77^a^ ± 2	70^b^ ± 1	0.019

^a, b^ The differences between the averages shown with different superscripts on the same line are statistically significant. DM = dry matter, CP = crude protein, CF = crude fiber, EE = ether extract, CD = commercial diet, CM = commercial moist diet, and CFM = commercial fermented moist diet (mean ± SD).

**Figure 1.**
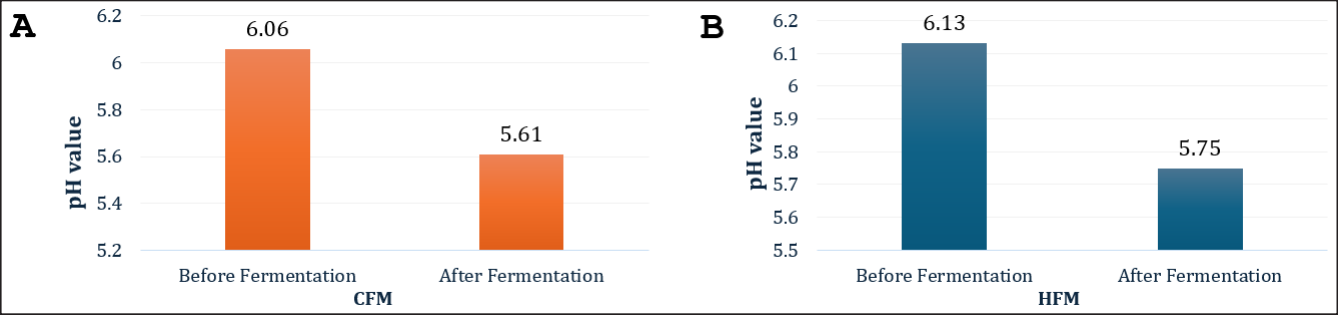
pH value before and after fermentation of the commercial (A) and hand-mixed (B) diets. CFM = commercial fermented moist, HFM = hand-mixed fermented moist.

In measuring fermentation efficiency, pH is an important parameter. In the commercial feeding trial, fermentation lowered the feed pH. Before fermentation, the pH value was 6.06, which reduced to 5.61 after fermentation (Figure 1A).

Economic analysis (Table 5) demonstrated that feed cost per kg live weight increased progressively from 0.80 USD (CD) to 0.86 USD (CFM) (*p* = 0.007), while production cost rose from 1.04 USD to 1.15 USD (*p* = 0.001). Consequently, profit per kg live weight declined significantly (*p* = 0.001) from 0.40 USD in CD to 0.20 USD in CFM.

**Table 5.** Economic performance of commercial diet in USD.

Parameters (%)	CD	CM	CFM	*p*-value
Feed cost/kg LW	0.80^b^ ± 0.01	0.82^b^ ± 0.02	0.86^a^ ± 0.01	0.007
Production cost/kg LW	1.04^c^ ± 0.01	1.09^b^ ± 0.03	1.15^a^ ± 0.01	0.001
Profit/kg LW	0.40^a^ ± 0.01	0.35^b^ ± 0.03	0.20^c^ ± 0.01	0.001

^a, b, c^ The differences between the averages shown with different superscripts on the same line are statistically significant. CD = commercial diet, CM = commercial moist diet, and CFM = commercial fermented moist diet; (Mean ± SD).

### 3.2. Trial 02 (Hand-mixed diet)

The proximate composition of hand-mixed diets (Table 6) showed clear enhancement with fermentation. CP content increased significantly (*p* = 0.002) from 22.8% in HM to 24.3% in HFM and 23.8% in HFM-SAA, whereas CF significantly decreased from 7.0% to 5.7% (*p* < 0.001). EE improved from 7.9% to 8.6% in HFM (*p* = 0.021), and ash content also significantly increased from 7.7% to 9.1% (*p* = 0.001). Conversely, NFE (nutrient-free energy) declined from 54.7% to 52.5% (*p* = 0.009). ME values increased significantly from 3426 to 3471 kcal/kg (*p* = 0.002), but the absence of synthetic amino acid remained statistically similar (3431 kcal/kg).

**Table 6.** Proximate Composition of Hand-mixed Diet (gm/100 gm DM).

Parameters (%)	HM	HFM	HFM-SAA	*p*-value
Crude protein	22.8^b^ ± 0.4	24.3^a^ ± 0.2	23.8^a^ ± 0.2	0.002
Crude fiber	7.0^a^ ± 0.1	5.7^b^ ± 0.2	5.9^b^ ± 0.1	< 0.001
Ether extract	7.9^b^ ± 0.3	8.6^a^ ± 0.3	8.1^ab^ ± 0.1	0.021
Ash	7.7^b^ ± 0.3	8.8^a^ ± 0.1	9.1^a^ ± 0.2	0.001
NFE	54.7^a^ ± 1	52.5^b^ ± 0.4	53.1^b^ ± 0.1	0.009
ME (kcal/kg)	3426^b^ ± 5	3471^a^ ± 12	3431^b^ ± 10	0.002

^a, b^ The differences between the averages shown with different superscripts on the same line are statistically significant. HM = hand-mixed moist diet, HFM = hand-mixed fermented moist diet, HFM-SAA = hand-mixed fermented moist diet without synthetic amino acid, NFE = nitrogen-free extract, and ME = metabolic energy (mean ± SD).

Fermentation had a highly significant effect on growth performance (Table 7). Birds receiving HFM reached the highest FBW (1446 gm) and BWG (1327 gm), compared with 1319 gm in HM and 1235 gm in HFM-SAA, with highly significant variation (*p* < 0.001). ADWG followed a similar pattern, with HFM achieving 47.4 gm/day, significantly higher than HM (42.8 gm/day) and HFM-SAA (39.9 gm/day). FI was also significantly higher in HFM (2413 gm; *p* = 0.002), while FCR values remained statistically similar (*p* > 0.05) among treatments.

**Table 7.** Effect of feeding a yeast-fermented, hand-mixed diet on growth performance and feed efficiency (*n* = 48).

Parameters (%)	HM	HFM	HFM-SAA	*p*-value
IBW	119 ± 2	119 ± 2	119 ± 2	1.000
FBW	1319^b^ ± 17.0	1446^a^ ± 14.4	1235^c^ ± 16.7	< 0.001
BWG	1199^b^ ± 18.6	1327^a^ ± 16.0	1116^c^ ± 17.6	< 0.001
ADBW	42.8^b^ ± 00.7	47.4^a^ ± 00.6	39.9^c^ ± 0.63	< 0.001
FI	2281^b^ ± 79.3	2413^a^ ± 6.11	2168.9^b^ ± 10.3	0.002
FCR	1.90 ± 0.09	1.82 ± 0.03	1.94 ± 0.04	0.104

^a, b, c^ The differences between the averages shown with different superscripts on the same line are statistically significant IBW = initial body weight; FBW = final body weight; BWG = body weight gain; ADWG = average daily weight gain; FI = feed intake (kg); FCR = feed conversion ratio (mean ± SD).

Digestibility data (Table 8) showed significant enhancements in DM (84%) and CP (80%) digestibility for HFM compared to HM (81% and 76%) and HFM-SAA (78% and 72%) (*p* ≤ 0.01). Digestibility of CF (crude fiber) and EE (ether extract) did not differ significantly.

**Table 8.** DM, CP, CF, and EE digestibility of hand-mixed diet.

Parameters (%)	HM	HFM	HFM-SAA	*p*-value
Digestibility DM	81^b^ ± 1	84^a^ ± 1	78^c^ ± 1	< 0.001
Digestibility CP	76^b^ ± 1	80^a^ ± 1	72^c^ ± 1	0.001
Digestibility CF	11 ± 1	8 ± 2	8 ± 2	0.204
Digestibility EE	69 ± 3	73 ± 3	69 ± 2	0.217

^a, b, c^ The differences between the averages shown with different superscripts on the same line are statistically significant. DM = dry matter, CP = crude protein, CF = crude fiber, EE = ether extract, HM = hand-mixed moist diet, HFM = hand-mixed fermented moist diet, HFM-SAA = hand-mixed fermented moist diet without synthetic amino acid (mean ± SD).

The pH of the hand-mixed fermented feed also declined from 6.13 before fermentation to 5.75 after fermentation (Figure 1B).

The economic outcomes (Table 9) clearly favored the fermented hand-mixed diet. Although feed cost was statistically non-significant across treatments (*p* = 0.094), production cost was lowest in HFM (1.27 USD/kg LW) compared to HM (1.35 USD) and HFM-SAA (1.40 USD; *p* = 0.009). Consequently, profit per kg live weight is highest at 0.17 USD in HFM, nearly double that of HM (0.09 USD) and significantly higher than HFM-SAA (0.04 USD; *p* = 0.009).

**Table 9.** Economic performance of hand-mixed diet in USD.

Parameters (%)	HM	HFM	HFM-SAA	*p-* value
Feed cost/kg LW	0.94 ± 0.04	0.90 ± 0.01	0.95 ± 0.02	0.094
Production cost/kg LW	1.35^ab^ ± 0.05	1.27^b^ ± 0.02	1.40^a^ ± 0.03	0.009
Profit/kg LW	0.09^ab^ ± 0.05	0.17^a^ ± 0.02	0.04^b^ ± 0.03	0.009

^a, b^ The differences between the averages shown with different superscripts on the same line are statistically significant. HM = hand-mixed moist diet; HFM = hand-mixed fermented moist diet; and HFM-SSA = hand-mixed moist diet after fermentation but without synthetic amino acids (mean ± SD).

## 4. Discussion

### 4.1. Trial-01 (Commercial diet)

Fermentation did not substantially alter the proximate composition of the commercial feed, consistent with the findings that the fortified formulation already contained processed ingredients and additives that limited microbial activity [19, 21]. A small numerical variation was observed during fermentation, but it did not enhance nutrient concentration or availability in this feed type. Here, the pH of the fermented commercial feed has declined, confirming active microbial activity; Aktar et al. [29] also demonstrate that fermentation reduced the feed’s pH.

Birds fed the fermented commercial diet (CFM) had lower final body weight and higher FCR, suggesting a negative effect of fermentation on feed palatability or nutrient integrity. Qiu et al. [19] noted that a higher level of additives in the feed formulation can reduce beneficial microbial growth during fermentation, which may explain the limited effect observed in this trial. Similar reductions in performance were observed when fortified commercial feeds were fermented [21]. FI reduction in the moist and fermented diets further supports earlier findings that wet feeding may be less accepted by broilers [10, 30]. Moreover, large-scale wet feeding remains impractical because it often provides no clear nutritional advantage [31].

The reduction in DM and EE digestibility in CFM is consistent with the findings of Chiang et al. [32] and Hafeez et al. [33], who reported that over-fermentation can oxidize or bind lipids to microbial biomass, thereby lowering fat digestibility.

Economically, fermentation increased feed and production costs, including the additional costs of yeast and the fermentation process. These results suggest that for additive-rich commercial feeds already optimized with enzymes, amino acids, and antioxidants, further fermentation may not provide value and could reduce performance by altering feed stability or palatability.

### 4.2. Trial-02 (Hand-mixed diet)

Fermentation markedly reduced CF and increased CP, which reflect microbial enzyme activity that breaks down cell wall polysaccharides and releases bound amino acids. Similar compositional improvements were reported by Azrinnahar et al. [17] and Abdel-Raheem et al. [34], who attributed them to increased proteolysis and carbohydrate hydrolysis during yeast fermentation. In pH value changes, a similar result was observed in trial-01, which supports the findings of Aktar et al. [29].

The higher FI in HFM suggests that fermentation increased palatability, likely due to the mild acidity and aroma produced during fermentation [35, 36], but it is lower in HFM-SAA, which may result from elevated fiber content [37], further confirming the necessity of synthetic amino acid supplementation in feed.

The improvements in DM and CP digestibility are consistent with findings from Predescu et al. [35], Katu et al. [10], and Sawant et al. [38], who demonstrate that fermented feed enhances nutrient absorption by reducing anti-nutritional factors and promoting beneficial gut microbiota. Although gut morphology was not measured in this study, improved digestibility likely reflects increased villus height and Lactobacillus proliferation, as widely reported for yeast-fermented diets [39, 40].

Performance dropped sharply in HFM-SAA, confirming that fermentation does not replace the need for synthetic amino acids. This is consistent with Al-Khalaifah et al. [22] and Hafeez et al. [33], who found that amino acid supplementation remains essential even when fermentation improves nutrient digestibility. Economically, the HFM treatment yielded the lowest production cost and the highest profit. Similar cost benefits were reported by Ezema and Eze [25] and Isikwenu et al. [41], who found that fermentation enhanced feed efficiency and reduced the cost per kilogram of live weight in small-scale broiler systems.

### 4.3. Comparative response between commercial and hand-mixed diets

The dissimilar results between the two feeding systems highlight that the benefits of fermentation are perspective-dependent. In additive-rich commercial diets, fermentation likely altered the stability of vitamins, enzymes, and synthetic amino acids, causing lower performance. In simpler hand-mixed diets, fermentation served as a biological enrichment process by enhancing nutrient digestibility and utilization. This supports the conclusions by Katu et al. [10] and Missotten et al. [21], who emphasized that fermentation is most effective when applied to unfortified or minimally processed diets.

## 5. Conclusions

Fermentation using *S. cerevisiae* improves nutrient digestibility and profitability in additive-free hand-mixed feeds. However, its use in complex commercial feeds should be optimized to prevent performance reduction. The findings also revealed that synthetic amino acids remain essential even in fermented diets, as their absence lowered nutrient utilization and performance. This study was limited to a single fermentation duration, yeast level, and moisture content, and it did not assess gut microbial responses. Future research should optimize fermentation conditions and evaluate nutrient stability and intestinal microbiota to clarify the practical application of fermented feeds in broiler nutrition.

## Data Availability

The data presented in this study are available from the corresponding author upon reasonable request.

## List of abbreviations

°C, degree Celsius; ADWG, average daily weight gain; BWG, body weight gain; CF, crude fiber; CP, crude protein; DM, dry matter; EE, ether extract; FBW, final body weight; FCR, feed conversion ratio; FI, feed intake; gm, gram; gm/day, gram per day; kcal/kg, kilocalories per kilogram; kg, kilogram; ME, metabolizable energy; MT, metric ton; SAA, synthetic amino acid.
